# POSTN knockdown suppresses IL‐1β‐induced inflammation and apoptosis of nucleus pulposus cells via inhibiting the NF‐κB pathway and alleviates intervertebral disc degeneration

**DOI:** 10.1002/ccs3.12030

**Published:** 2024-05-07

**Authors:** Zhaoheng Wang, Daxue Zhu, Fengguang Yang, Haiwei Chen, Jihe Kang, Wenzhao Liu, Aixin Lin, Xuewen Kang

**Affiliations:** ^1^ Department of Orthopedics Lanzhou University Second Hospital Lanzhou China; ^2^ Key Laboratory of Orthopedics Disease of Gansu Province Lanzhou University Second Hospital Lanzhou China

**Keywords:** apoptosis, inflammation, intervertebral disc degeneration, NF‐κB pathway, nucleus pulposus, POSTN

## Abstract

The aim of this study is to investigate the effects of POSTN on IL‐1β induced inflammation, apoptosis, NF‐κB pathway and intervertebral disc degeneration (IVDD) in Nucleus pulposus (NP) cells (NPCs). NP tissue samples with different Pfirrmann grades were collected from patients with different degrees of IVDD. Western blot and immunohistochemical staining were used to compare the expression of POSTN protein in NP tissues. Using the IL‐1β‐induced IVDD model, NPCs were transfected with lentivirus‐coated si‐POSTN to down‐regulate the expression of POSTN and treated with CU‐T12‐9 to evaluate the involvement of NF‐κB pathway. Western blot, immunofluorescence, and TUNEL staining were used to detect the expression changes of inflammation, apoptosis and NF‐κB pathway‐related proteins in NPCs. To investigate the role of POSTN in vivo, a rat IVDD model was established by needle puncture of the intervertebral disc. Rats were injected with lentivirus‐coated si‐POSTN, and H&E staining and immunohistochemical staining were performed. POSTN expression is positively correlated with the severity of IVDD in human. POSTN expression was significantly increased in the IL‐1β‐induced NPCs degeneration model. Downregulation of POSTN protects NPCs from IL‐1β‐induced inflammation and apoptosis. CU‐T12‐9 treatment reversed the protective effect of si‐POSTN on NPCs. Furthermore, lentivirus‐coated si‐POSTN injection partially reversed NP tissue damage in the IVDD model in vivo. POSTN knockdown reduces inflammation and apoptosis of NPCs by inhibiting NF‐κB pathway, and ultimately prevents IVDD. Therefore, POSTN may be an effective target for the treatment of IVDD.

## Graphical abstract

In the present experiment, the mechanism of action of POSTN on IL‐1β‐induced nucleus pulposus cells was investigated. Inhibition of POSTN expression can alleviate the inflammatory response and apoptosis of nucleus pulposus cells, and this effect is mediated by inhibiting the activity of NF‐κB pathway. It is believed that our study contributes to the elucidation of the pathogenesis of intervertebral disc degeneration and raises the awareness of the role of POSTN in intervertebral disc degeneration. At the same time, a new perspective on the potential treatment of intervertebral disc degeneration is presented.

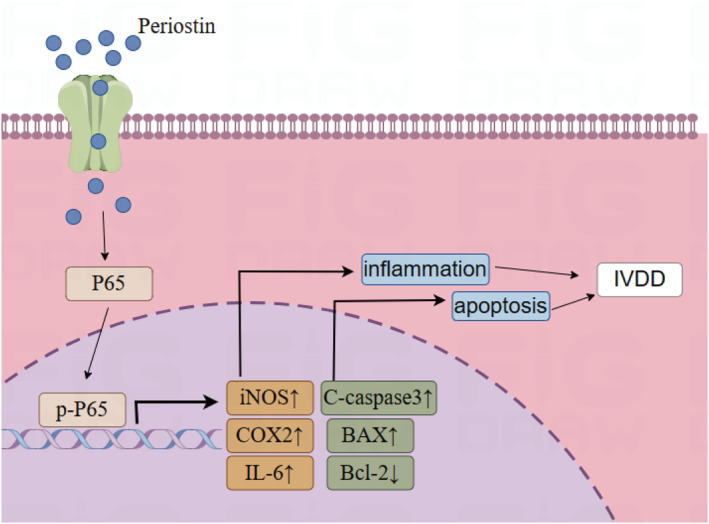

## 1 INTRODUCTION

Lower back pain represents a significant societal and public health challenge, affecting approximately 11%–84% of the global population throughout their lifetime. This condition is closely linked to lumbar intervertebral disc degeneration (IVDD), which stands as one of the primary contributors to lumbar disc degeneration, thereby imposing substantial detriments on both quality of life and economic well‐being for individuals and society.^1^, ^2^, ^3^, ^4^ Epidemiological studies have demonstrated an age‐related increase in the prevalence of lumbar disc degeneration within society.^5^ IVDD is a complex ailment influenced by various factors, including environmental, nutritional, and genetic aspects,^6^ which contribute to cellular morphological alterations, inflammation, augmented senescent cells, apoptosis, and autophagy. Presently available treatments for IVDD primarily encompass conservative or surgical approaches; however, these are merely symptomatic interventions with limited potential to effectively decelerate or halt the progression of IVDD.^1^ Consequently, there exists an urgent imperative to discover novel therapeutic agents targeting the mitigation of discogenic degeneration.^7^

The intervertebral disc, a fibrocartilaginous tissue situated between two vertebrae, comprises three interconnected structures: the central nucleus pulposus (NP), the peripheral annulus fibrosus, and the cartilaginous endplates on the upper and lower surfaces.^8^ The NP, a semi‐transparent gelatinous tissue, serves as the fundamental component of the disc that upholds spinal column stability.^3^ Nucleus pulposus cells (NPCs) primarily consist of an extracellular matrix abundant in type II collagen, elastin, and proteoglycans. During spinal motion, NPs' spherical structure effectively disperses pressure while supporting extensive‐angle and high‐frequency movements to facilitate physiological activities throughout the rest of the spine.^9^ IVDD is a multifaceted cell‐mediated process, wherein diverse external and internal stimuli induce senescence, apoptosis, and inflammation in NPCs, thereby compromising the normal biological function of discs and ultimately leading to IVDD.^6^ Inflammation and apoptosis of NPCs within the intervertebral disc are widely acknowledged as the primary etiological factors contributing to IVDD.^10^, ^11^, ^12^ During the course of IVDD, there is a substantial upregulation of inflammatory factors in degenerating disc cells. The heightened expression levels of these inflammatory mediators, including IL‐1α, IL‐1β, IL‐6, and TNF‐α, result in a reduction in NPCs population along with long‐term deterioration of the microenvironment.^13^, ^14^, ^15^ Furthermore, a substantial quantity of inflammatory factors accelerates the degradation process of proteoglycans and type II collagen within the extracellular matrix. Consequently, an elevation in degradation products from the extracellular matrix triggers the production of inflammatory mediators, thereby leading to further deterioration of this matrix.^16^, ^17^ Additionally, disruption in the internal environment of the extracellular matrix can excessively activate abnormal apoptosis in NPCs. Furthermore, abnormal apoptosis in NPCs can also induce disruption within the extracellular matrix itself. This subsequently exacerbates the progression of IVDD.^18^ Therefore, investigating both inflammation and apoptosis mechanisms within NPCs will provide valuable insights into understanding IVDD pathogenesis and aid in identifying potential therapeutic targets.

IL‐1β, extensively investigated as an inflammatory factor associated with IVDD,^14^ not only facilitates extracellular matrix degradation,^19^ pyroptosis,^20^ and oxidative stress^21^ in NPCs, but also predominantly triggers the production of multiple inflammatory factors in NPCs, thereby exacerbating the inflammatory response.^22^, ^23^ Moreover, IL‐1β induces the synthesis of pro‐apoptotic proteins while inhibiting the expression of anti‐apoptotic proteins, thus contributing to aberrant apoptosis of intervertebral disc cells.^24^ Furthermore, Kim et al. have reported that the induction of homogeneous neuropathic pain and inflammatory responses can be achieved by puncturing a rat intervertebral disc and injecting a specific concentration of IL‐1β at the time of puncture.^25^ Given the crucial role played by IL‐1β in the pathomechanism of IVDD, it becomes imperative to explore effective strategies for mitigating IL‐1β‐induced inflammatory and apoptotic reactions while restoring the equilibrium between anabolic and catabolic activities of NPCs in order to proactively prevent disc degeneration onset.

NF‐κB, a class of transcription factors, assumes pivotal roles in numerous physiological and pathological processes. The NF‐κB pathway can be categorized into two distinct pathways: the classical and non‐classical pathways.^26^ It is widely acknowledged that activation of the classical NF‐κB pathway exerts crucial regulatory functions in inflammation, immune response, cell proliferation, differentiation, and survival.^26^, ^27^ The NF‐κB pathway plays a crucial role in maintaining the internal environment homeostasis of the intervertebral disc.^28^, ^29^ It is noteworthy that the NF‐κB pathway stands out as one of the prominent signaling pathways activated during IVDD, with NPCs inflammation, catabolism, and mechanical loading being its primary stimulatory factors.^30^ Sun et al.^31^ discovered that BAY11‐7082, a specific inhibitor of the NF‐κB pathway, effectively suppressed IL‐1β‐induced activation of NF‐κB. Furthermore, inhibition of the NF‐κB pathway resulted in reduced expression levels of MMP‐3, MMP‐9, MMP‐13, ADAMTS‐4, and ADAMTS‐5 induced by IL‐1β while promoting the up‐regulation of proteoglycan and type II collagen expression. Additionally, activation of the NF‐kB pathway was found to enhance the production of inflammatory mediators such as nitric oxide synthase (iNOS) and cyclooxygenase (COX‐2). These enzymes generate nitric oxide and induce prostaglandin expression which hinders proteoglycan formation and collagen type II synthesis within the NP leading to extracellular matrix disorganization.^32^ The NF‐κB pathway can also inhibit apoptosis induced by excessive mechanical stretch stress and enhance the viability of NPCs through lentiviral transfection‐mediated inhibition of P65. Inhibition of NF‐κB effectively mitigates inflammation and degeneration in NPCs associated with IVDD.^33^ Notably, recent studies have revealed intricate interactions between NF‐κB and non‐coding RNAs that may further exacerbate or delay the onset and progression of IVDD.^30^ For instance, in degenerated NP tissues, the augmented expression levels of TNF‐α and IL‐1β stimulate the upregulation of miR‐640, which subsequently amplifies NF‐κB signaling pathway activity. This establishes a positive feedback loop that culminates in heightened levels of inflammatory factors within the NP microenvironment, thereby expediting NP degeneration.^34^ Consequently, the pivotal role played by NF‐κB pathway activity in IVDD necessitates targeting this pathway as an efficacious therapeutic approach.^35^

Periostin (POSTN) is an extracellular matrix protein that plays a crucial role in regulating the microenvironment and modulating cellular behavior as well as tissue homeostasis.^36^ It exhibits low expression levels in most normal tissues, while being highly expressed in various inflammatory disorders such as asthma,^37^ allergy,^38^ pulmonary fibrosis,^39^ and cancers.^40^, ^41^ An immunolocalization study of 11 lumbar intervertebral disc specimens obtained through surgery or donation revealed the presence of POSTN in the cytoplasm of both the inner and outer annulus fibrosus, as well as in certain cells within the NP.^42^ Notably, a majority of disc cells exhibited immunoreactivity, particularly in the outer annulus. Quantitative analysis demonstrated that the percentage of POSTN‐positive cells was significantly higher in the outer annulus (88.8%) compared to the inner annulus (61.4%), while expression levels were lowest within the NP with an average rate of only 18.5%. Notably, in a separate investigation, Tsai et al.^43^ employed gene chip technology to acquire gene expression profiles of disc degeneration and conducted PCR analysis to determine the expression levels of degeneration‐related markers such as POSTN. The findings revealed elevated mRNA and protein levels of the POSTN gene in human degenerating NPCs. Furthermore, histologic examination demonstrated significantly higher positive staining for POSTN in degenerated disc tissue compared to non‐degenerated disc tissue. However, contradicting previous discoveries, a study investigating aberrantly expressed genes during senescence in rat NPCs^44^ reported down‐regulated expression of POSTN in degenerating disc tissues. In a recent study, Zhu et al.^45^ discovered that POSTN plays a crucial role in promoting apoptosis and exacerbating degeneration of intervertebral disc cells through the activation of the Wnt/β‐catenin pathway. Consequently, this present investigation aims to further elucidate the intricate relationship between POSTN and degenerated intervertebral discs.

In addition, Chijimatsu et al.^46^ discovered that POSTN induced an upregulation of MMP‐1, MMP‐3, MMP‐13, IL‐6, IL‐8, and NOS2 in human chondrocytes leading to osteoarthritis (OA). They further demonstrated that the induction of POSTN in this study was mediated by the nuclear translocation of P65 and subsequent activation of the NF‐κB pathway. In a separate investigation on temporomandibular joint osteoarthritis, Fan et al.^47^ observed that POSTN triggered the nuclear translocation of P65 which subsequently influenced ADAMTS5 expression. Henceforth, it is plausible to suggest a correlation between POSTN and the NF‐κB pathway in both the initiation and progression of disc degeneration. Moreover, through activating the NF‐κB pathway effectively induces inflammation and apoptosis within NPCs thereby promoting disc degeneration onset.

In our study, we aimed to investigate the potential of lentivirus‐mediated small interfering RNA transfection in inhibiting POSTN expression and subsequently reversing IL‐1β‐induced inflammatory response and abnormal apoptosis in NPCs. The objectives of this research were twofold: (1) to further validate the detrimental effects of POSTN on NPCs during disc degeneration, and (2) to establish a novel theoretical framework for identifying molecular targets that could be targeted for therapeutic interventions against disc degeneration.

## 2 RESULT

### 2.1 POSTN expression is increased in human degenerative NP tissues

**FIGURE 1:**
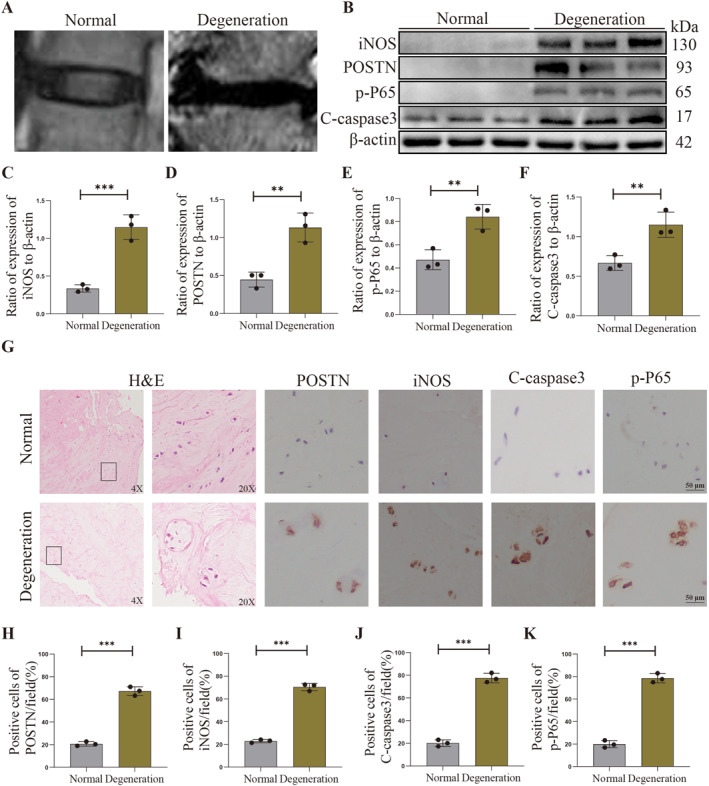
POSTN expression is increased in human degenerative nucleus pulposus (NP) tissues. (A) MRI of the spine from patients with different Pfirrmann grades. (B–F) Protein expression levels and quantitative analysis of POSTN, p‐P65, iNOS and C‐caspase3 in normal and degenerated human NP tissues by Western Blot. (G–K) H&E staining of normal and degenerated human NP tissues. Expression levels and quantitative analysis of POSTN, p‐P65, iNOS and C‐caspase3 in normal and degenerated human NP tissues by immunohistochemical staining. *p* < 0.05 indicates statistical significance, *p < 0.05, **p < 0.01, ***p < 0.001. These values are expressed as means ± SD of at least three independent experiments.

POSTN is widely expressed in various tissues, and its aberrant expression has been implicated in numerous diseases, including cancer and inflammatory disorders. To establish a definitive association between POSTN and the progression of human IVDD, we examined normal and degenerated human NP tissues to ascertain the levels of POSTN expression. Additionally, we aimed to investigate the correlation between the expression of iNOS, an inflammatory factor, pro‐apoptotic protein C‐caspase3, NF‐κB p‐P65, and the development of IVDD. Following Pfirmann grading assessment, we carefully selected normal and degenerated human NP tissues for further analysis by dividing them into two groups comprising six cases each. We chose human NP tissues from patients with different grades of degeneration according to Pfirrmann grading by MRI examination (Figure 1A). Figure 1G displays the H&E staining of normal and degenerated NP tissues, revealing a well‐organized arrangement of collagen fibers and a homogeneous extracellular matrix in non‐degenerated NP tissues. Conversely, degenerated NP tissues exhibited significant aggregation of NPCs, evident fibrosis, disarrayed collagen fibrils, and a notable reduction in the extracellular matrix. Additionally, immunohistochemical staining was conducted to assess the expression levels of POSTN, p‐P65, iNOS, and C‐caspase3 in both normal and degenerated human NP tissues. The results demonstrated a substantial increase in the expression levels of POSTN, p‐P65, iNOS, and C‐caspase3 within degenerated NP tissues compared to their normal counterparts (*p* < 0.05, Figure 1G–K). Subsequently, we conducted Western Blot assays on both normal and degenerated NP tissues, revealing a significant up‐regulation of POSTN, p‐P65, iNOS and C‐caspase3 protein levels in the latter compared to the former (*p* < 0.05, Figure 1B–F), which was consistent with our immunohistochemical findings. These results provide further evidence for the involvement of these proteins in IVDD.

### 2.2 POSTN expression is increased in rat degenerative NP tissues

**FIGURE 2:**
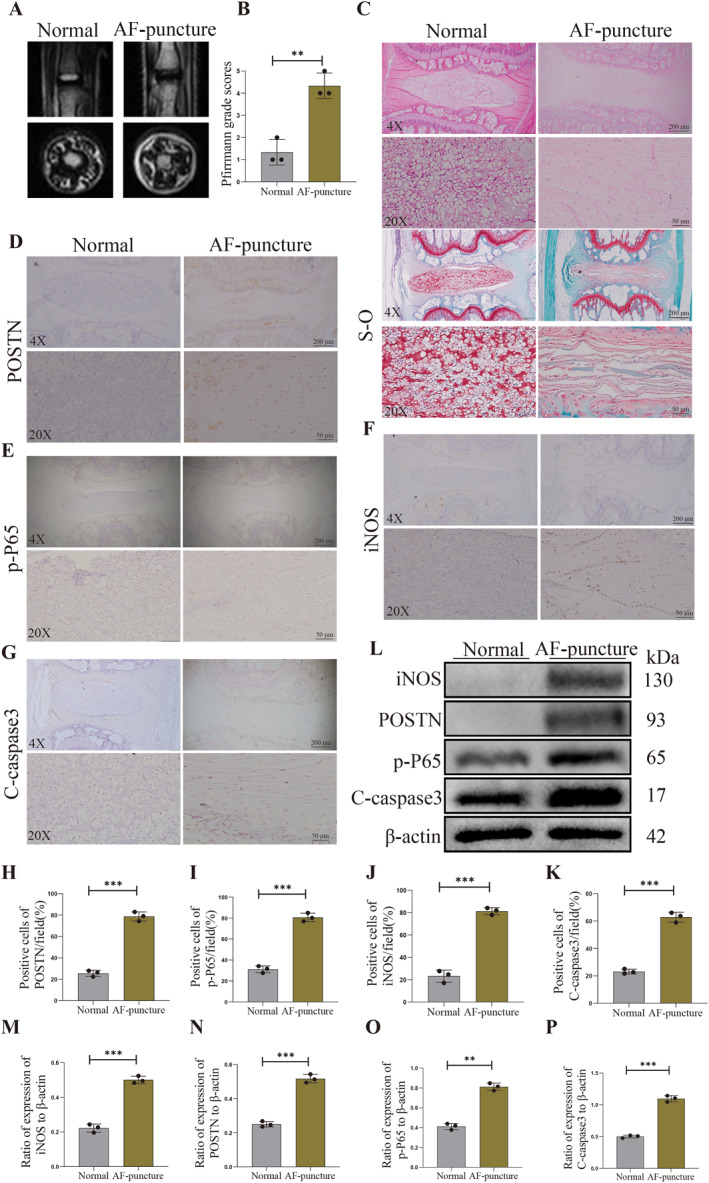
POSTN expression is increased in rat degenerative nucleus pulposus (NP) tissues. (A and B) MRI images and Pfirrmann score in rat discs of control and AF puncture groups. (C) H&E and Safranin‐O/fast green staining of normal and degenerated rat NP tissues. (D–K) Immunohistochemical staining of POSTN, p‐P65, iNOS and C‐caspase3 expression in normal and degenerated rat NP tissues and quantitative analysis. (L–P) Protein expression levels and quantitative analysis of POSTN, p‐P65, iNOS and C‐caspase3 in normal and degenerated human NP tissues were detected by Western Blot. *p* < 0.05 indicates statistical significance, *p < 0.05, **p < 0.01, ***p < 0.001. These values are expressed as at least three independent experimental means ± SD are indicated.

In the present study, we have confirmed a significant upregulation of POSTN, p‐P65, iNOS and C‐caspase3 expression in degenerated human NP tissues compared to normal NP tissues. To further investigate the association between these markers and rat NP tissue degeneration, we established a rat model of NP tissue degeneration using fine‐needle puncture into the caudal vertebrae. A standardized histopathology scoring system for IVDD in rat models was used to evaluate the model. The MRI results indicated that the T2 image water signal of rat disc without acupuncture (control) was higher than that of the AF puncture group (Figure 2A), and the Pfirrmann grade score in the AF puncture group was higher than that in the control (Figure 2B). Figure 2C depicts H&E staining and Safranin‐O/fast green staining of normal rat NP tissues and those that were needled to induce degeneration. Our findings provide valuable insights into the molecular mechanisms underlying intervertebral disc degeneration and may pave the way for novel therapeutic strategies targeting these markers. Based on the H&E staining and Safranin‐O/fast green staining results, it was observed that the fibers of rat NP tissues in the normal group exhibited a well‐organized alignment and a uniformly abundant extracellular matrix. In contrast, compared to the normal group, degenerated rat NP tissues induced by needling displayed disorganized fiber arrangement along with a significant reduction in NPCs and an evident disruption of the extracellular matrix. These findings from H&E staining and Safranin‐O/fast green staining provide compelling evidence for the successful establishment of the rat IVDD model using acupuncture method. Subsequently, we conducted immunohistochemical staining to investigate the differential expression of POSTN, p‐P65, iNOS, and C‐caspase3 in the normal group and the acupuncture degeneration group. The immunohistochemical staining revealed a significant increase in the expression levels of POSTN, p‐P65, iNOS, and C‐caspase3 in the NP tissues of degenerated rats compared to those in the normal group (*p* < 0.05, Figure 2D–K). Next, we conducted Western Blot analysis to assess the protein levels of POSTN, p‐P65, iNOS, and C‐caspase3 in both groups. The experimental findings revealed a significant upregulation of POSTN, p‐P65, iNOS, and C‐caspase3 protein expression in the acupuncture degeneration group compared to the normal group (*p* < 0.05, Figure 2L‐P). These results are consistent with those obtained through immunohistochemical staining.

### 2.3 POSTN expression is increased in IL‐1β‐induced NPCs degeneration model

**FIGURE 3:**
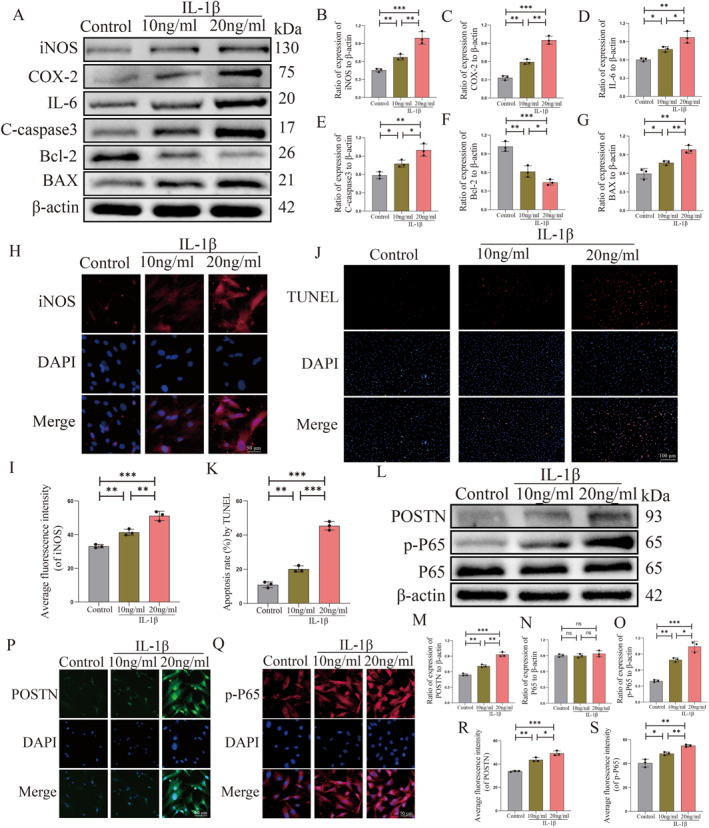
POSTN expression is increased in IL‐1β‐induced nucleus pulposus cells (NPCs) degeneration model. (A–G) Western Blot detection of iNOS, COX‐2, IL‐6, C‐caspase3, BAX and Bcl‐2 expression and quantitative analysis in control and rat NPCs with IL‐1β concentrations of 10 ng/mL and 20 ng/mL. (H and I) Immunofluorescence detection of fluorescence intensity and quantitative analysis of iNOS in control and rat NPCs with IL‐1β concentrations of 10 ng/mL and 20 ng/mL. (J and K) TUNEL staining for detection of apoptosis and quantitative analysis in control and rat NPCs with IL‐1β concentrations of 10 ng/mL and 20 ng/mL. (L–O) Western Blot to detect the expression of POSTN, P65 and p‐P65 in control and rat NPCs with IL‐1β concentrations of 10 ng/mL and 20 ng/mL and quantitative analysis. (P–S) Immunofluorescence assay for fluorescence intensity and quantitative analysis of POSTN and p‐P65 in control and rat NPCs with IL‐1β concentrations of 10 ng/mL and 20 ng/mL. *p* < 0.05 indicates statistical significance, *p < 0.05, **p < 0.01, ***p < 0.001. These values are expressed as means ± SD of three independent experiments at least.

Through our experiments, we have observed a significant increase in the expression of POSTN, p‐P65, iNOS and C‐caspase3 in degenerated NP tissues when compared to normal human and rat NP tissues. In order to further investigate whether this expression pattern is consistent with that of NPCs, we utilized IL‐1β as an intervention group to induce NPCs degeneration. To validate the accuracy and reliability of our established rat NPCs degeneration model, we intervened NPCs with concentrations of 10 ng/mL and 20 ng/mL IL‐1β for 24 h based on previous studies.^3^ We employed Western Blot analysis to assess the expression levels of inflammatory factors (iNOS, COX‐2, IL‐6) and pro‐apoptosis‐related proteins (C‐caspase3, BAX)/anti‐apoptosis‐related protein (Bcl‐2) in the control group as well as at IL‐1β concentrations of 10 ng/mL and 20 ng/mL. Our findings revealed a significant upregulation in the expression of iNOS, COX‐2, IL‐6, C‐caspase3, and BAX in response to IL‐1β intervention compared to the control group. Additionally, we observed a notable decrease in the expression of anti‐apoptosis‐related protein Bcl‐2. This finding provides evidence for the successful establishment of our IL‐1β degeneration model. Furthermore, we observed that the degeneration of rat NPCs was more pronounced in the IL‐1β intervention group at a concentration of 20 ng/mL compared to the group treated with 10 ng/mL (*p* < 0.05, Figure 3A–G). Additionally, immunofluorescence revealed an upregulation in iNOS expression with increasing concentrations of IL‐1β in both control and experimental groups (*p* < 0.05, Figure 3H–I). Consistent results were obtained from TUNEL staining, Western Blot analysis, and immunofluorescence (*p* < 0.05, Figure 3J–K), confirming a concentration‐dependent increase in NPCs apoptosis and further validating the success of our established degeneration model. In conclusion, our established model effectively simulates the degeneration of NPCs by utilizing IL‐1β concentrations of 10 ng/mL and 20 ng/mL to represent distinct grades of NPCs. This reliable model can be utilized for further investigations into the correlation between POSTN and p‐P65 expression with IVDD, as well as for subsequent studies in this field.

**FIGURE 4:**
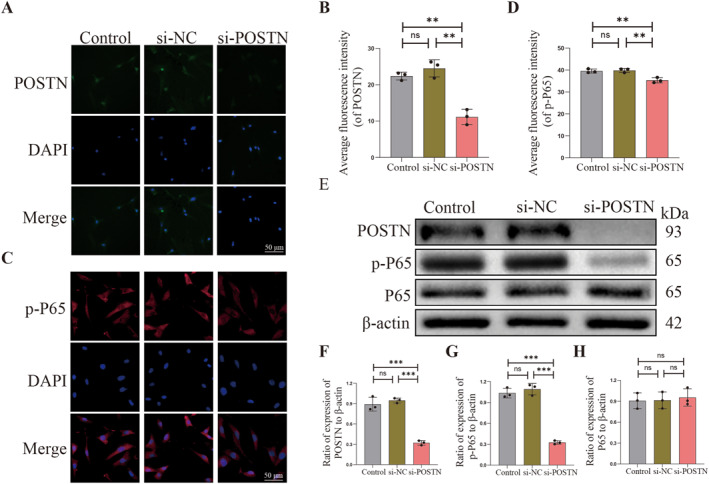
Successful transfection of rat nucleus pulposus cells by si‐POSTN inhibits POSTN expression. (A–D) Immunofluorescence detection of POSTN and p‐P65 in control, si‐NC and si‐POSTN groups and quantitative analysis. (E–H) Western Blot detection of POSTN, P65 and p‐P65 protein expression and quantitative analysis in control, si‐NC and si‐POSTN groups. *p* < 0.05 indicates statistical significance, *p < 0.05, **p < 0.01, ***p < 0.001. These values are expressed as means ± SD of at least three independent experiments expressed.

Subsequently, we conducted Western Blot to assess the expression levels of POSTN and NF‐κB P65 as well as p‐P65 in control rat NPCs and those treated with IL‐1β concentrations of 10 ng/mL and 20 ng/mL. Our findings revealed a positive correlation between the degree of degeneration in rat NPCs and the upregulation of POSTN and p‐P65 expression (*p* < 0.05, Figure 3L‐O). Interestingly, the expression level of P65 remained consistent irrespective of the increasing degree of degeneration observed in rat NPCs. These findings offer valuable insights into the molecular mechanisms underlying NPCs degeneration. Furthermore, we also investigated the immunofluorescence expression of POSTN and p‐P65, revealing a corresponding increase in fluorescence intensity with escalating IL‐1β concentration. Notably, our immunofluorescence results for p‐P65 demonstrated that NF‐κB P65 was inert and did not undergo nuclear translocation in the control group without any intervention; however, upon intervention with varying concentrations of IL‐1β on rat NPCs, P65 exhibited significant activation and subsequent nuclear translocation (*p* < 0.05, Figure 4P–S). We observed that IL‐1β not only elicited an upregulation in POSTN expression, but also triggered the activation of the NF‐κB pathway and subsequent phosphorylation of P65, leading to its translocation into the nucleus. In summary, our findings demonstrate an augmented expression of POSTN, p‐P65, iNOS and C‐caspase3 in the rat model of NPCs degeneration.

### 2.4 POSTN knockdown suppresses IL‐1β‐induced inflammation and apoptosis of NPCs

**FIGURE 5:**
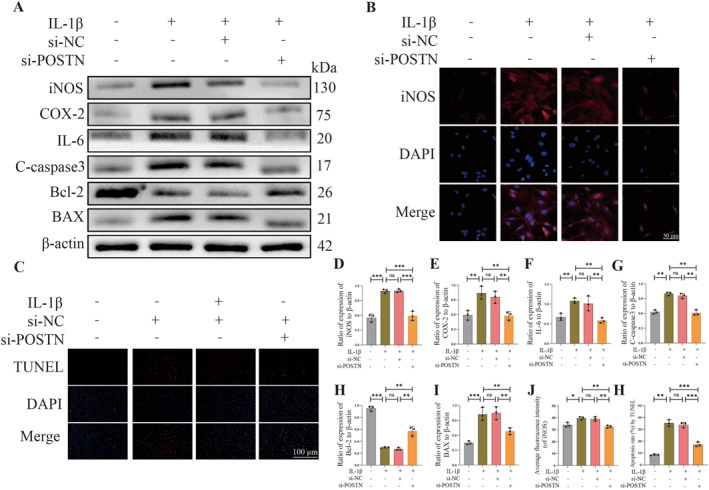
POSTN knockdown suppresses IL‐1β‐induced inflammation and apoptosis of nucleus pulposus cells (NPCs). (A, D–I) Western Blot detection of iNOS, COX‐2, IL‐6, C‐caspase3, BAX, and Bcl‐2 expression and quantitative analysis in Control, IL‐1β, IL‐1*β*+si‐NC, and IL‐1*β*+si‐POSTN groups. (B and J) Immunofluorescence detection of iNOS fluorescence intensity in each group and quantitative analysis. (C and H) TUNEL staining to detect apoptosis of rat NPCs in each group and quantitative analysis. *p* < 0.05 indicates statistical significance, *p < 0.05, **p < 0.01, ***p < 0.001. These values are expressed as means ± SD of at least three independent experiments.

The aforementioned experiments have demonstrated a positive correlation between the expression of POSTN and the degree of degeneration in rat NPCs, suggesting that POSTN may play a pivotal role in NPCs degeneration. Previous studies^45^ have reported that POSTN exhibits both time‐dependent and concentration‐dependent effects on rat NPCs degeneration. By subjecting rat NPCs to recombinant POSTN at concentrations of 1 μg/mL, 5 μg/mL, and 10 μg/mL for a 24‐h incubation period, it was observed through Western Blot, TUNEL staining, and flow cytometry that POSTN promotes concentration‐dependent degeneration of rat NPCs. Subsequently, following the selection of recombinant POSTN at the same concentration (5 μg/mL) for co‐culturing with rat NPCs for 24, 48, and 72 h, it was observed that the degeneration of NPCs increased proportionally with longer exposure to recombinant POSTN. This finding clearly demonstrates the time‐dependent effect of POSTN on NPCs degeneration. To further investigate changes in NPCs degeneration resulting from inhibited expression of POSTN, we transfected rat NPCs with lentivirus carrying small interfering RNAs (si‐NC and si‐POSTN). Subsequently, we established control groups consisting of unaltered NPCs and NPCs transfected with an empty vector si‐NC to assess successful transfection through Western Blot and immunofluorescence. Western Blot results demonstrated that the expression of POSTN, P65, and p‐P65 remained unaffected in NPCs not transfected with lentiviruses. However, a significant suppression in the expression of POSTN and p‐P65 was observed in NPCs transfected with si‐POSTN (*p* < 0.05, Figure 5E–H). Furthermore, immunofluorescence revealed a substantial reduction in fluorescence intensity for both POSTN and p‐P65 in si‐POSTN‐transfected NPCs (*p* < 0.05, Figure 5A–D), indicating the effective inhibition of POSTN expression by lentiviral‐mediated delivery of small interfering RNAs. Consequently, our successful transfection of rat NPCs has been confirmed.

After successful transfection of rat NPCs with lentivirus‐mediated small interfering RNA (siRNA), we divided the NPCs into four groups to investigate the impact of POSTN inhibition on inflammatory response and apoptotic levels in degenerated NPCs. These groups included: Control group, IL‐1β group, IL‐1β + si‐NC group, and IL‐1β + si‐POSTN group. In the IL‐1β group and IL‐1β + si‐POSTN group, we supplemented 20 ng/mL of IL‐1β to simulate NPC degeneration. We investigated the differential expression of inflammatory factors iNOS, COX‐2, and IL‐6 as well as pro‐apoptosis‐related proteins (C‐caspase3, BAX) and anti‐apoptosis‐related protein (Bcl‐2) in subgroups using Western Blot analysis. The Western Blot results demonstrated a significant upregulation of iNOS, COX‐2, IL‐6, C‐caspase3, and BAX expression compared to the Control group when exposed to IL‐1β or IL‐1*β*+ si‐NC. Conversely, the expression of Bcl‐2 was downregulated in these groups. Notably, there were no significant differences observed in the expression levels of iNOS, COX‐2, IL‐6, C‐caspase3, BAX, and Bcl‐2 between the two experimental groups: IL‐1β group and IL‐1β + si‐NC group. However, successful transfection of si‐POSTN in the IL‐1β group resulted in a significant reversal of the aforementioned factors (*p* < 0.05, Figure 5A,D‐I). Immunofluorescence of iNOS expression in each subgroup also confirmed these findings, with significantly stronger fluorescence intensity observed in the IL‐1β and IL‐1β + si‐NC groups compared to the Control group. No significant difference was observed between these two groups; however, the fluorescence intensity of iNOS was significantly lower in the IL‐1β + si‐POSTN group compared to both the IL‐1β and IL‐1β + si‐NC groups (*p* < 0.05, Figure 5B,J). In addition, the TUNEL staining results were found to be consistent with the Western Blot findings (*p* < 0.05, Figure 5C,H), thereby reinforcing the notion that inhibition of POSTN gene expression significantly mitigates the inflammatory response and apoptosis levels in degenerating rat NPCs. These experimental outcomes further underscore the pivotal role played by POSTN in rat NPC degeneration.

### 2.5 POSTN knockdown protects the inflammation and apoptosis of NPCs by inhibiting the NF‐κB pathway

Our previous experiments have demonstrated a significant upregulation of NF‐κB p‐P65 expression in degenerated NP tissues compared to undegenerated NP tissues, both in humans and rats. Furthermore, we observed an increase in the expression level of p‐P65 protein with the degree of degeneration in rat NPCs using an IL‐1β‐induced degeneration model. Additionally, when we transfected NPCs with lentivirus‐carried small interfering RNA to inhibit POSTN expression, Western Blot revealed a significant suppression of p‐P65 expression upon inhibition of POSTN. Hence, our findings suggest a substantial interaction between POSTN and NF‐κB pathways in IVDD, collectively contributing to the degeneration process within the NP.

**FIGURE 6:**
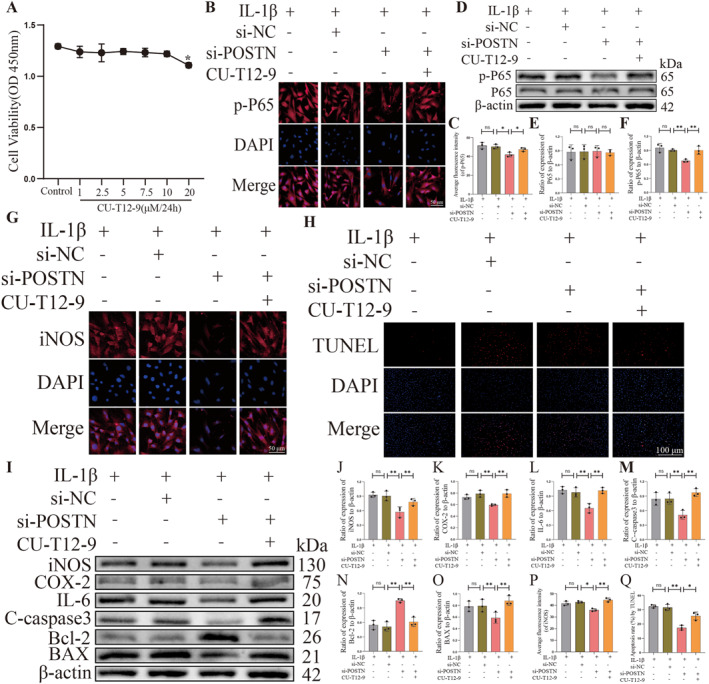
POSTN knockdown protects the inflammation and apoptosis of nucleus pulposus cells (NPCs) by inhibiting the NF‐κB pathway. (A) Cell viability assay to detect the effects of different concentrations of CU‐T12‐9 on the viability of normal rat NPCs. (B and C) Immunofluorescence to detect the fluorescence intensity and quantitative analysis of P65 and p‐P65 in each group. (D–F) Western Blot detection of P65 and p‐P65 expression in each group and quantitative analysis. (G and P) Immunofluorescence detection of fluorescence intensity and quantitative analysis of iNOS in each group. (H and Q) TUNEL staining for apoptosis of rat NPCs in each group and quantitative analysis. (I–O) Western Blot to detect the expression of iNOS, COX‐2, IL‐6, C‐caspase3, BAX, and Bcl‐2 in each group and quantitative analysis. *p* < 0.05 indicates statistical significance, *p < 0.05, **p < 0.01, ***p < 0.001. These values are expressed as means ± SD of at least three independent experiments.

To this end, we aim to investigate the impact of inhibiting the POSTN gene on the activity of the NF‐κB pathway. We divided rat NPCs into four groups: IL‐1β group, IL‐1β + si‐NC group, IL‐1β + si‐POSTN group, and IL‐1β + si‐POSTN + CU‐T12‐9 group. Subsequently, we employed Western Blot and immunofluorescence to assess the differential expression of NF‐κB P65 and p‐P65. CU‐T12‐9 serves as an agonist for the NF‐kB pathway by promoting TLR1 and TLR2 dimerization, thereby leading to increased downstream inflammatory factors such as TNF‐a and IL10. To determine the impact of varying concentrations of CU‐T12‐9 on rat NPCs viability, we conducted a 24‐h co‐culture experiment using different concentrations of CU‐T12‐9 (0, 1, 2.5, 5, 7.5, 10 and 20 μM) in a 96‐well plate setup. The CCK‐8 assay was employed to assess the effects of these concentrations on NPCs viability. Our findings revealed no significant alteration in NPCs viability within the range of CU‐T12‐9 concentrations from 0 to 10 μM; however, an observable decline in cell viability was observed when the concentration reached up to 20 μM (*p* < 0.05, Figure 6A). The Western Blot results demonstrated that the viability of NPCs in the IL‐1β + si‐POSTN group remained unaffected, as compared to both the IL‐1β group and the IL‐1β + si‐NC group. Moreover, there was a significant reduction in NF‐κB p‐P65 protein expression, indicating that si‐POSTN effectively inhibited the activity of the NF‐κB signaling pathway. Interestingly, in the presence of CU‐T12‐9 (IL‐1β + si‐POSTN + CU‐T12‐9 group), we observed a reversal of the initial decreasing trend of p‐P65 levels, with a subsequent increasing trend (*p* < 0.05, Figure 6D–F). Immunofluorescence revealed that transfection with si‐POSTN suppressed P65 activation and its nuclear translocation in IL‐lβ‐induced NPCs. However, upon addition of CU‐T12–9, we observed a significant increase in fluorescence intensity and activation of P65 along with nuclear translocation (*p* < 0.05, Figure 6B–C).

Our study has demonstrated that inhibition of POSTN gene expression leads to a reduction in the activity of the NF‐κB pathway. However, upon addition of CU‐T12‐9, this effect is reversed and P65 is activated for nuclear translocation. In order to further validate whether POSTN regulates the inflammatory response of NPCs through the NF‐κB pathway, we investigated the variations in iNOS, COX‐2, IL‐6, C‐caspase3, BAX, and Bcl‐2 expression levels within each group using Western Blot and immunofluorescence. We categorized the cells into four groups: the IL‐1β group, the IL‐1β + si‐NC group, the IL‐1β + si‐POSTN group, and the IL‐1β + si‐POSTN + CU‐T12‐9 group. The Western Blot results demonstrated that in comparison to the IL‐1*β*+si‐POSTN group, there was an elevated expression of iNOS, COX‐2, IL‐6, C‐caspase3, BAX and Bcl2 in the IL 1β + si‐POSTN group. Notably, caspase3 and BAX exhibited a significant reduction compared to both preceding groups; however, Bcl2 displayed a substantial increase. Interestingly though when CU‐T12‐9 was introduced to these groups, there was a reversal observed in the aforementioned indices (*p* < 0.05; Figure 6I–O). The immunofluorescence results also demonstrated a significant increase in the fluorescence intensity of iNOS within the IL‐1β + si‐POSTN + CU‐T12‐9 group compared to the IL‐1β + si‐POSTN group (*p* < 0.05, Figure 6G,P). This suggests that inhibition of POSTN expression attenuated the activity of the NF‐κB pathway, thereby ameliorating the inflammatory response in degenerating NPCs. Similarly, we also examined whether POSTN regulates NPCs apoptosis levels through the NF‐κB pathway, and the TUNEL staining results were consistent with those obtained from Western Blot (*p* < 0.05, Figure 6H,Q).

## 3 DISCUSSION AND CONCLUSION

In this study, we present multiple lines of evidence supporting the expression profile of POSTN in NP tissues and its involvement in IL‐1β‐induced inflammation and apoptosis in NPCs. Specifically, our findings demonstrate that: (1) POSTN expression is upregulated in degenerating NPs, and lentiviral‐mediated inhibition of POSTN reverses the inflammatory and apoptotic responses induced by IL‐1β; (2) suppression of POSTN expression attenuates NPCs inflammation and apoptosis by inhibiting P65 nuclear translocation, thereby blocking NF‐κB pathway. Taken together, these results suggest that targeting POSTN may hold promise as a therapeutic strategy for disc degeneration.

IL‐1β plays a crucial role in various pathological changes associated with IVDD and is considered a pivotal mediator of IVDD and lower back pain.^14^ Importantly, IL‐1β not only induces the expression of matrix metalloproteinases (MMPs) and A Disintegrin And Metalloproteinase with Thrombospondin motifs (ADAMTSs), leading to disruption of the extracellular matrix,^48^, ^49^ but also exacerbates the inflammatory response of NPCs by stimulating the secretion of several pro‐inflammatory factors, including iNOS, COX‐2, IL‐6, and TNF‐α^24^, ^50^ Furthermore, IL‐1β‐induced apoptosis and pyroptosis in NPCs are significant contributors to disc degeneration.^20^, ^24^ These pathological responses ultimately contribute to the development of disc degeneration. In order to investigate the impact of IL‐1β on NPCs degeneration, we conducted in vitro experiments following a previously established research method.^51^ Rat NPCs were intervened with varying concentrations of IL‐1β to establish rat NPCs models representing different grades of degeneration. Based on experimental results, cells treated with IL‐1β concentrations of 10 ng/mL and 20 ng/mL were utilized to model distinct levels of degeneration. Western blot revealed that the expression levels of inflammatory factors such as iNOS, COX‐2, and IL‐6 increased proportionally with the degree of cellular degeneration. Furthermore, it was observed that upregulation in inflammatory factor expression was mediated by IL‐1β through activation of the NF‐κB pathway. IL‐1β can also induce aberrant apoptosis in NPCs through activation of the NF‐κB pathway, leading to upregulation of pro‐apoptosis‐related proteins C‐caspase3 and BAX, as well as downregulation of the anti‐apoptosis‐related protein Bcl‐2 in a dose‐dependent manner. Our findings underscore the crucial role played by IL‐1β in IVDD, highlighting its significance for potential therapeutic interventions.

POSTN, an extracellular matrix protein, has been increasingly implicated in the pathogenesis and progression of various diseases.^36^, ^52^, ^53^ As a well‐established biomarker, POSTN plays a pivotal role in diverse orthopedic conditions.^46^, ^47^, ^54^ Recent literature also highlights that recombinant POSTN can activate the Wnt/β‐catenin pathway to upregulate BAX and C‐caspase3 expression while downregulating Bcl‐2 expression. This activation ultimately leads to apoptosis of NPCs and subsequent IVDD. Furthermore, lentiviral‐mediated inhibition of POSTN expression resulted in decreased BAX and C‐caspase3 levels along with increased Bcl‐2 expression.^45^ Although this study has demonstrated the prodegenerative effect of POSTN on NPCs, it did not address the protective effect of inhibiting POSTN expression on NPCs under IL‐1β intervention. In our study, we first examined the expression of POSTN in human and rat NP tissues as well as in rat NPCs of different degeneration grades. We found that POSTN was not only expressed at an increased level in degenerating NP, but also that the expression level of POSTN protein increased significantly with increasing degeneration grade, which is consistent with previous studies.^45^ This suggests that POSTN is likely to play a pivotal role in the initiation and progression of disc degeneration, highlighting its significance as a key molecular factor. Moreover, upon silencing POSTN expression in NPCs through lentiviral transfection, we observed a remarkable reversal of the pro‐inflammatory and pro‐apoptotic effects induced by IL‐1β. Western blot analysis revealed a significant decrease in the expression levels of iNOS, COX‐2, IL‐6, C‐caspase3, and BAX while an increase was observed in Bcl‐2 expression within IL‐1β‐treated NPCs following inhibition of POSTN. Consistent results were obtained through immunofluorescence and TUNEL assay. Furthermore, our investigation utilizing Western Blot and immunofluorescence revealed that the inhibition of POSTN expression exhibited a significant suppressive effect on the nuclear translocation of P65. Consequently, this led to a notable reduction in the activity of the NF‐κB pathway. These findings align consistently with previous studies investigating the intricate relationship between POSTN and the NF‐κB pathway in OA.^46^, ^47^

The NF‐κB pathway, which is intricately associated with inflammation and apoptosis, has been demonstrated to play a crucial role in IVDD.^30^ In a recent investigation by Cao et al.,^55^ it was reported that inhibition of the NF‐κB pathway led to reprogramming of the energy metabolism in NPCs. Furthermore, Zheng et al.^56^ exhibited that inhibiting the NF‐κB pathway effectively mitigated the endoplasmic reticulum stress response induced by IL‐1β in NPCs. Zheng et al.^57^ have reported that inhibition of the NF‐κB pathway can alleviate inflammation‐related pain in a disc degeneration model. Our study has demonstrated that suppression of POSTN expression can reverse the pro‐inflammatory and pro‐apoptotic effects induced by IL‐1β, while also reducing the activity of the NF‐κB pathway. Therefore, we hypothesize that POSTN may play a role in disc degeneration by activating the NF‐κB pathway to induce inflammation and apoptosis in NPCs. These findings suggest potential therapeutic targets for treating IVDD. First, we confirmed through Western Blot and immunohistochemistry that the expression of NF‐κB p‐P65 was upregulated in degenerated NP tissues from both human and rat subjects. Additionally, we observed an increase in the protein levels of p‐P65 in rat NPCs with varying degrees of degeneration. Subsequently, we conducted pretreatment experiments on rat NPCs using IL‐1β, lentivirus, and CU‐T12‐9, a specific agonist of TLR1/2 known to activate NF‐κB pathway. Notably, CU‐T12‐9 induced an elevation in downstream effectors such as TNF‐α, IL‐10, and iNOS.^58^ The Western Blot results revealed that CU‐T12‐9 exhibited a significant activating effect on NF‐κB in NPCs, leading to the inhibition of POSTN expression. Additionally, it up‐regulated the expression of inflammatory factors and pro‐apoptosis‐related proteins while down‐regulating the expression of anti‐apoptosis‐related proteins. These findings were further confirmed by Immunofluorescence and TUNEL staining. Consequently, this study demonstrates that POSTN induces inflammation and apoptosis in NPCs through activation of the NF‐κB pathway. Importantly, these results align with previous research indicating that POSTN can activate the NF‐κB pathway to promote inflammation in chondrocytes, thereby exacerbating OA.^46^

Our study demonstrated that lentiviral transfection effectively inhibited the expression of POSTN, leading to a significant reduction in IL‐1β‐induced inflammatory response and aberrant apoptosis in NPCs. In recent years, numerous studies have consistently reported a strong association between POSTN expression and IL‐1β across various tissues.^59^, ^60^, ^61^, ^62^ In a study investigating the bioinformatics analysis of key regulators involved in synoviocyte migration and invasion in rheumatoid arthritis, You et al.^63^ discovered that POSTN, identified as a potential crucial regulator of synoviocyte invasiveness, exhibited elevated expression levels in synoviocytes affected by rheumatoid arthritis, indicating a distinct pro‐inflammatory signal. This increased expression of POSTN was specifically induced by IL‐1β. Furthermore, IL‐13, recognized as a pivotal molecule in type II inflammation,^64^ has been extensively documented to stimulate the production of POSTN across various cell types. Consequently, POSTN functions as an important downstream mediator of IL‐13 with diverse biological activities. Therefore, we postulate that IL‐1β may elicit the upregulation of POSTN expression in chondrocytes or chondrocyte‐like cells. The potential correlation between POSTN and IL‐1β could serve as a promising therapeutic avenue for chronic inflammatory disorders such as rheumatoid arthritis and intervertebral disc NP degeneration. Regrettably, there remains a paucity of research elucidating the underlying mechanisms governing the interaction between POSTN and IL‐1β.

Although our study confirmed the upregulation of POSTN expression in degenerating NP and demonstrated that inhibiting POSTN expression could reverse IL‐1β‐induced inflammation and apoptosis in NPCs by attenuating NF‐κB pathway, it is important to acknowledge certain limitations in our study. Firstly, IVDD involves not only the NP but also the annulus fibrosus and cartilaginous endplates. The relationship between POSTN and the annulus fibrosus and cartilaginous endplates remains unclear, necessitating further research to elucidate its association with other intervertebral disc cells. Additionally, we employed the pinprick method to model IVDD in rats; however, it is important to note that there are notable disparities in the mechanical environment between rat and human intervertebral discs. Therefore, further investigation necessitates the utilization of more scientifically advanced modeling methods or conducting more extensive animal studies. Additionally, it is worth noting that there exists substantial evidence indicating a significant correlation between POSTN and IL‐1β, suggesting their potential significance as targets in intervertebral disc degeneration development. However, this aspect was not thoroughly explored within the scope of this study, thus highlighting its importance for future research endeavors.

**FIGURE 7:**
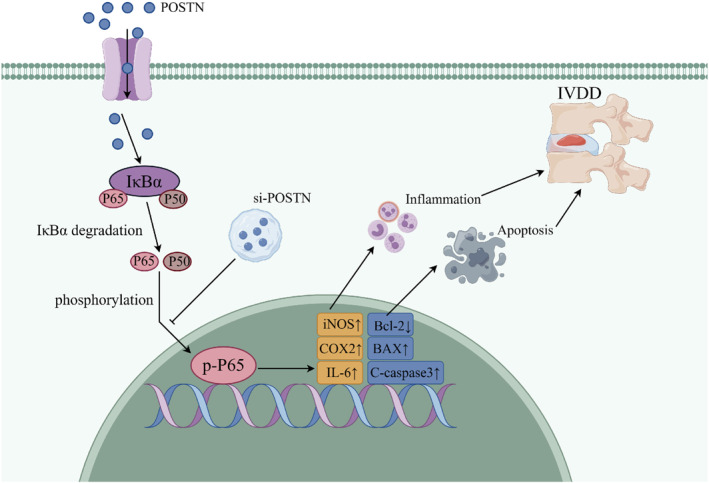
Schematic diagram illustrating that POSTN knockdown suppresses IL‐1β‐induced inflammation and apoptosis of nucleus pulposus cells via inhibiting the NF‐κB pathway and alleviates intervertebral disc degeneration.

In summary, the expression of POSTN exhibited an upward trend in both human and rat intervertebral disc NP tissues as the severity of disc degeneration increased. In vitro experiments demonstrated that inhibition of POSTN expression effectively delayed disc degeneration by modulating the NF‐κB pathway and reversing IL‐1β‐induced inflammation and apoptosis in NPCs (Figure 7). These findings highlight the potential of targeting POSTN as a molecular intervention for treating disc degeneration, thus providing valuable insights for future therapeutic strategies.

## 4 MATERIALS AND METHODS

### 4.1 Isolation and culture of rat NPCs

After male Sprague Dawley rats (180–220 g, 4 weeks old) were executed with an overdose of pentobarbital (100 mg/kg) under aseptic conditions, their tails were cut off with tissue scissors and skin was peeled off, and the caudal vertebrae were rapidly transferred to an ultra‐clean operating table after being immersed in 75% alcohol for 1 min. The gelatinous NP tissue of the tail was aseptically dissected using a sterile scalpel and tissue forceps. The isolated NP tissues were exposed to 0.25% type II collagenaseor for at least 2 h at 37°C. However, the study described that NP tissues were incubated with 0.25% type II collagenase at 37°C for 45 minutes.^5^ So, NP tissue was completely digested after incubating with 0.25% type II collagenase at 37°C for 30 min.^65^, ^66^, ^67^, ^68^, ^69^ The digestion process was terminated by adding basal medium to the mixture. Following centrifugation (1200 r, 5 min), the supernatant was discarded, and pre‐configured DMEM/F12 complete medium was added and thoroughly mixed using a pipette gun before transferring it to T25 culture flasks. Subsequently, the cultures were incubated at 37°C and 5% CO2 in an incubator. The complete medium was replaced when most cells were observed adhering to the flask wall under microscopic examination, thereafter changing the medium every 3 days. When the cells reached 90% confluence under microscopic observation, a solution of trypsin containing 0.25% EDTA was introduced to facilitate isolation and subsequent passaging for culture. NP cells from generations 2‐4 were utilized for subsequent experimental procedures.

### 4.2 Treatment of rat NPCs

Rat NPCs in the logarithmic growth phase were collected and adjusted to a concentration of 5 × 10^5^/ml in T25 cell vials. They were then inoculated into 5 mL DMEM/F12 medium supplemented with 13.7% fetal bovine serum and cultured in a cell culture incubator (37°C, 5% CO2) for 24 h. Upon reaching a confluence degree of 70%, the cells were treated with different concentrations of IL‐1β (10 ng/mL, 20 ng/mL) individually for another 24 h. Alternatively, after pretreatment with lentivirally transfected si‐POSTN or si‐NC, the cells were cultured until they reached a confluence degree of up to 70% in the presence or absence of IL‐1β (20 ng/mL) and the NF‐κB agonist CU‐T12‐9 (10 mM). Subsequently, protein extraction was performed. DMSO was used as the solvent for solubilizing the agonist.

### 4.3 Western Blot

The rat NPCs were pre‐treated and subsequently washed three times with cold PBS. For cell protein lysis, RIPA lysis solution containing protease inhibitor and phosphatase inhibitor was added. The T25 cell vials were gently shaken every 5 min on ice to ensure complete cell lysis. After 20 min, the cells were observed under a microscope to confirm full lysis. If complete cell lysis was observed, the lysate was transferred to a centrifuge tube and centrifuged at 12,000 r for 20 min at 4°C. Following centrifugation, the supernatant was supplemented with pre‐configured sample buffer and boiled at 100°C. Rat and human NP tissue proteins were extracted using the same procedure described above. The protein concentration of the boiled samples was determined using the BCA method.

Equal amounts of proteins were separated using 10% fast gel electrophoresis at a voltage of 120 V, followed by transfer onto a PVDF membrane. Subsequent membrane transfer was conducted at a current of 350 mA. After successful membrane transfer, the PVDF membranes were incubated with 5% skimmed milk powder for 1 h at room temperature and then further incubated overnight at 4°C with primary antibodies including POSTN (1:1000, Abcam), iNOS (1:1000, Proteintech), COX‐2 (1:2000, Abcam), IL‐6 (1:1000, Immunoway), C‐caspase3 (1:1000, Proteintech), BAX (1:1000, Proteintech), Bcl‐2 (1:1000, Proteintech), P65 (1:1000, Immunoway), p‐P65 (1:1000, Immunoway) and *β*‐actin (1:2000, Proteintech). The following day, the PVDF membranes were washed three times with TBST. Subsequently, the membranes were incubated with horseradish peroxidase‐labeled secondary antibodies for 1 h; rabbit secondary antibody was used at a dilution of 1:5000 and mouse secondary antibody was used at a dilution of 1:70,000. Finally, protein bands were detected using enhanced chemiluminescence reagents after an additional three washes with TBST.

### 4.4 Immunofluorescence

When the cells reached 90% confluence, they were trypsinized with 0.25% EDTA and the resulting NPCs were seeded onto coverslips in six‐well plates at a density of 1 × 10^5^ before being placed in an incubator. Upon reaching 80% confluence, the cells were washed thrice with PBS for 5 minutes each time, fixed with 4% paraformaldehyde for 20 minutes at room temperature, and then washed again thrice with oscillating PBS for 5 minutes each time. Subsequently, the cells were permeabilized using 0.2% Triton X‐100 for 30 minutes at room temperature followed by three washes with oscillating PBS lasting five minutes each time. 10% goat serum was added to each coverslip in the six‐well plate for incubation. Subsequently, 10% goat serum was applied to seal each coverslip of the six‐well plate. After incubating at 37°C for 1 h, the surface of the specimen was covered with primary antibodies (POSTN at a dilution of 1:300, iNOS at a dilution of 1:300, and p‐P65 at a dilution of 1:300) and left overnight at 4°C. The following day, specimens were rewarmed for 1 hour at 37°C, washed three times with PBS for 5 minutes per wash, and then incubated with FITC‐labeled fluorescent secondary antibody for 1 hour while being protected from light. Nuclei were stained using fresh DAPI solution under light protection before blocking and observation under a fluorescence microscope followed by image capture.

### 4.5 Lentiviral transfection

The expression of POSTN was suppressed by transfection of lentivirus carrying the inhibitory target gene. Following the reagents and instructions provided by GENE, and considering the growth characteristics of rat NPCs, we determined a virus transfection efficiency MOI = 100 through pre‐experimentation. Based on the virus titer provided by GENE and the previously obtained MOI value, we transfected rat NPCs with an empty vector transfected group (si‐NC) that did not inhibit POSTN expression, as well as a lentivirus transfected group (si‐POSTN) capable of suppressing POSTN expression. Subsequently, we separately transfected rat NPCs with an empty vector transfection group (si‐NC), which lacked inhibition toward POSTN, and a lentivirus transfection group (si‐POSTN), which effectively inhibited the expression of POSTN gene. To enhance the transfection effect of the lentivirus, we supplemented P or A transfection enhancement solution accordingly. After transfection, the cells were cultured in a cell culture incubator for 12 h. The success of transfection was assessed using fluorescence microscopy, following which the cells were replenished with pre‐configured complete medium and continued to be cultured in the same incubator. Upon reaching 90% confluence, the cells were passaged, rendering them suitable for subsequent experimental procedures.

### 4.6 Cell viability assay

To assess the impact of NF‐κB pathway activator CU‐T12‐9 on rat NPCs viability, we seeded 5 × 103 cells/well in 96‐well plates and followed CCK‐8 kit instructions for subsequent assay procedures. After cell attachment under a microscope, different concentrations of CU‐T12‐9 were administered to the cells for 24 h. The supernatant was aspirated and washed with PBS thrice before adding 10 μL of CCK‐8 solution per well and incubating at 37°C for an hour. Finally, absorbance was measured at 450 nm using an enzyme meter, repeating the experiment three times to obtain mean values for further analysis.

### 4.7 Immunohistochemical staining

Follow the instructions of the immunochromogenic kit. The collected NP tissues of human or rat were placed in 4% paraformaldehyde for fixation, in which the NP tissues of rat were also placed in decalcified solution for 1 week for decalcification treatment, and then the fixed NP tissues were routinely dehydrated, embedded, sliced, etc. The treated paraffin sections were placed in an incubator and baked at 70°C for 2 h. Then the sections were successively placed in xylene—xylene −100% alcohol −100% alcohol −95% alcohol −85% alcohol −75% alcohol for dewaxing treatment, including 12 min for xylene twice and 5 min for alcohol with different concentration gradients. Then the slices were washed in clean water for 2 min. Then the slices were placed in the antigen repair solution, and the antigen repair solution was heated at high heat for 3 min in the microwave oven to expose the antigen binding site. After the antigen repair solution was cooled to room temperature, endogenous peroxidase blocker was added to the area defined by the oil pen, and incubated at room temperature for 10 min. After washing with PBS for 3 times, non‐specific stain blocker was added and incubated at room temperature for 10 min. After the non‐specific stain blockers were removed, the primary antibodies were added: POSTN (1:300), iNOS (1:300), C‐caspase3 (1:300), p‐P65 (1:300) were incubated at 4°C overnight. On the second day, after rewarming for 1 h, PBS was washed 3 times, biotin‐labeled sheep anti‐rat/rabbit IgG polymer was added, and incubated at room temperature for 10 min. After washing with PBS for 3 times, *Streptomyces* antibioproteinase‐peroxidase was added and incubated at room temperature for 10 min. After washing with PBS for 3 times, the pre‐configured DAB color developing solution was used for incubation and color development. After rinsing with running water, the nucleus was stained with hematoxylin. After rinsing with running water, the slices were successively placed in 75% alcohol‐85% alcohol‐95% alcohol‐100% alcohol‐100% alcohol‐xylene xylene for dehydration treatment. Finally, neutral gum was used to seal the film and the image was collected under the optical microscope.

### 4.8 H&E staining

The procedure was performed according to the H&E staining kit instructions. The collected human or rat NP tissues were placed in 4% paraformaldehyde for fixation, in which the rat NP tissues were also placed in decalcification solution for decalcification for 1 week, and then the fixed NP tissues were routinely subjected to dehydration, embedding, sectioning and other treatments. The paraffin sections were baked in a thermostat at 70°C for 2 h. The sections were then sequentially deparaffinized in xylene‐xylene‐100% alcohol‐100% alcohol‐95% alcohol‐85% alcohol‐75% alcohol, with xylene in each of the two steps for 12 min, and different gradients of alcohol in each of the two steps for 5 min, and then the sections were washed in water for 2 min stained with hematoxylin staining solution for 10–20 min, washed clean with water and then differentiated with acid differentiation solution for 15 s, then washed clean with water and stained with eosin staining solution for 1–2 min. After washed clean with water, the stained sections were sequentially placed in 75% alcohol‐85% alcohol‐95% alcohol‐100% alcohol‐ 100% alcohol‐xylene‐ xylene for dehydration, and finally, the slices were sealed with Finally, the slices were sealed with neutral gum and images were acquired under a light microscope.

### 4.9 TUNEL staining

The TUNEL assay, known in full as Terminal Deoxynucleotidyl Transferase (TdT)‐mediated dUTP Nick end labeling, is used to detect DNA fragments after apoptosis. Briefly, cells are placed on glass cover slips in 6‐well culture plates, pretreated accordingly, and assayed according to the manufacturer's instructions. Under a fluorescence microscope, DNA fragments of post‐apoptotic cells are colored red by the Apoptosis Detection Kit.

### 4.10 Safranin‐O/fast green staining

To measure the extent of IVDD in human and rat discs, all rats were sacrificed 8 weeks after surgery, and disc tissue sections (5 mm) were cut for Safranin‐O/fast green (Solarbio) stains. The assay was performed according to the manufacturer's instructions. Images were captured using a light microscope (Olympus).

### 4.11 Establishment of rat IVDD model by puncture method

After the purchased SD rats were kept in the animal experiment center for 1 week, the establishment of rat IVDD model was carried out under aseptic conditions. Rats weighing about 180–220 g were taken, and they were randomly divided into two groups: the sham‐operated group and the needle‐prick degeneration group. The rats were first anaesthetized by intraperitoneal injection of 1% sodium pentobarbital at a dose of 40 mg/kg. The anaesthetized rats were placed in the prone position, and the caudal vertebrae were exposed by a scalpel incision along the middle of the tail of the rats under aseptic conditions, and the NP tissue was inserted with the needle of a 21G syringe parallel to the endplate of the cartilage, at a depth of 5 mm, and the needle was rotated and held for 15 s. The control group had ‐no needling intervention. Finally, the wounds were closed using silk sutures, and the rats were continued to be housed in the Animal Experimentation Center. Four weeks after surgery, the rats were euthanized by intraperitoneal injection of an overdose of sodium pentobarbital, and the NP tissues were collected and the specimens in each group were randomly divided into two. One copy was stored in liquid nitrogen for subsequent protein extraction from NP tissues, and the other was fixed and decalcified for subsequent H&E staining and immunohistochemical staining.

### 4.12 Statistical analysis

All data in this experiment were statistically analyzed by SPSS 25.0 (IBM, Armonk, NY, USA) software, and all experimental data were expressed as mean ± standard deviation, and each experiment was repeated independently at least three times. Differences between two groups were analyzed using the independent samples *t*‐test, and data differences between multiple groups were analyzed using one‐way analysis of variance (ANOVA). *p* < 0.05 was considered statistically significant.

## AUTHOR CONTRIBUTIONS

Zhaoheng Wang, Daxue Zhu and Fengguang Yang conceived and wrote the article and had equal contributions to the article. Zhaoheng Wang and Daxue Zhu contributed to making of figures. Zhaoheng Wang and Fengguang Yang wrote the manuscript. Zhaoheng Wang, Haiwei Chen, Jihe Kang, Wenzhao Liu, Aixin Lin revised the manuscript. Zhaoheng Wang and Xuewen Kang contributed to proofreading of the article. All authors read and approved the final manuscript.

## CONFLICT OF INTEREST STATEMENT

The authors declare no conflict of interest.

## ETHICS STATEMENT

This study was conducted in accordance with the Declaration of Helsinki, and approved by the Ethics Committee of the Lanzhou University Second Hospital.

## Data Availability

To protect the biological information and privacy of the donors of this study, the raw data are not to be shared publicly but are available on request from the authors.
